# The Antinociceptive Effects of JWH-015 in Chronic Inflammatory Pain Are Produced by Nitric Oxide-cGMP-PKG-KATP Pathway Activation Mediated by Opioids

**DOI:** 10.1371/journal.pone.0026688

**Published:** 2011-10-21

**Authors:** Roger Negrete, Arnau Hervera, Sergi Leánez, Jesús M. Martín-Campos, Olga Pol

**Affiliations:** 1 Grup de Neurofarmacologia Molecular, Institut de Recerca de l'Hospital de la Sta Creu i Sant Pau and Institut de Neurociències, Universitat Autònoma de Barcelona, Barcelona, Spain; 2 Grup de Bioquímica, Institut de Recerca de l'Hospital de la Sta Creu i Sant Pau, Barcelona, Spain; Universidad Federal de Santa Catarina, Brazil

## Abstract

**Background:**

Cannabinoid 2 receptor (CB2R) agonists attenuate inflammatory pain but the precise mechanism implicated in these effects is not completely elucidated. We investigated if the peripheral nitric oxide-cGMP-protein kinase G (PKG)-ATP-sensitive K^+^ (KATP) channels signaling pathway triggered by the neuronal nitric oxide synthase (NOS1) and modulated by opioids, participates in the local antinociceptive effects produced by a CB2R agonist (JWH-015) during chronic inflammatory pain.

**Methodology/Principal Findings:**

In wild type (WT) and NOS1 knockout (NOS1-KO) mice, at 10 days after the subplantar administration of complete Freund's adjuvant (CFA), we evaluated the antiallodynic (von Frey filaments) and antihyperalgesic (plantar test) effects produced by the subplantar administration of JWH-015 and the reversion of their effects by the local co-administration with CB2R (AM630), peripheral opioid receptor (naloxone methiodide, NX-ME) or CB1R (AM251) antagonists. Expression of CB2R and NOS1 as well as the antinociceptive effects produced by a high dose of JWH-015 combined with different doses of selective L-guanylate cyclase (ODQ) or PKG (Rp-8-pCPT-cGMPs) inhibitors or a KATP channel blocker (glibenclamide), were also assessed. Results show that the local administration of JWH-015 dose-dependently inhibited the mechanical and thermal hypersensitivity induced by CFA which effects were completely reversed by the local co-administration of AM630 or NX-ME, but not AM251. Inflammatory pain increased the paw expression of CB2R and the dorsal root ganglia transcription of NOS1. Moreover, the antinociceptive effects of JWH-015 were absent in NOS1-KO mice and diminished by their co-administration with ODQ, Rp-8-pCPT-cGMPs or glibenclamide.

**Conclusions/Significance:**

These data indicate that the peripheral antinociceptive effects of JWH-015 during chronic inflammatory pain are mainly produced by the local activation of the nitric oxide-cGMP-PKG-KATP signaling pathway, triggered by NOS1 and mediated by endogenous opioids. These findings suggest that the activation of this pathway might be an interesting therapeutic target for the treatment of chronic inflammatory pain with cannabinoids.

## Introduction

The activation of both cannabinoid receptors 1 (CB1R) and 2 (CB2R) reduce nociception in numerous animal pain models [1]–[3]. However, while the analgesic potential derived from the stimulation of CB1R is accompanied with several central site-effects, the administration of selective CB2R agonists reduces nociception without causing those effects [4]. As a consequence, the peripheral antinociceptive effects produced by selective CB2R agonists after local inflammation have been demonstrated in several works [2], [5]–[7]. It is well known that CB2R are mainly located in the peripheral nervous system, but although an increased expression of these receptors has been recently demonstrated in the dorsal root ganglia and paw of animals with acute (*2 hours*) peripheral inflammation [8], the probable changes in their peripheral expression after chronic inflammatory pain remains to be fully elucidated. Moreover, the possible mechanisms implicated in the peripheral actions of CB2R agonists during chronic inflammatory pain have not been evaluated.

Several studies have shown that nitric oxide, synthesized by neuronal nitric oxide synthase (NOS1), mediates numerous inflammatory pain symptoms [9]–[10] and the local antinociceptive effects of opioids during inflammation is mainly produced by the activation of the peripheral nitric oxide-cGMP-protein kinase G (PKG)-ATP-sensitive K^+^ (KATP) channels signaling pathway [11]–[13]. Recent studies also demonstrated that the activation of CB1R stimulates the cGMP production in neuronal cells [14], that the antinociceptive effects produced by a CB1 endocannabinoid are mainly mediated by the nitric oxide-cGMP pathway activation [15] and that the inactivation of the nitric oxide-cGMP-PKG peripheral pathway enhanced the peripheral antinociceptive effects of CB2R agonists during neuropathic pain [16]. Some works have been also shown that the antinociceptive effects produced by AM1241 (a specific CB2R agonists) were mediated through the release of endogenous opioid peptides from CB2R-expressing cells [17]–[18]. Even so, the participation of the local endogenous opioid peptides as well as the nitric oxide-cGMP-PKG-KATP signaling pathway in the peripheral antinociceptive effects produced by JWH-015 during chronic inflammatory pain is not known.

Thus, in order to study if the nitric oxide synthesized by NOS1 could modulate the local effects of CB2R agonists during chronic peripheral inflammation we evaluated the mechanical antiallodynic and thermal antihyperalgesic effects of the subplantar administration of 2-methyl-1-propyl-1H-indol-3-yl)-1-naphthalenylmethanone (JWH-015), in wild type (WT) and NOS1-KO mice, at 10 days after the complete Freund's adjuvant (CFA)-injection. The receptor specificity of these effects and the possible participation of the peripheral endogenous opioids in the effects produced by JWH-015 after inflammatory pain were assessed by evaluation their reversion with specific a CB2R (6-iodo-2-methyl-1-[2-(4-morpholinyl)ethyl]-1H-indol-3-yl](4-methoxyphenyl)methanone; AM630), a CB1R (*N*-(Piperidin-1-yl)-5-(4-iodophenyl)-1-(2,4-dichlorophenyl)-4-methyl-1*H*-pyrazole-3-carboxamide; AM251) or a peripherally acting opioid receptor (naloxone methioide; NX-ME) antagonist. The mRNA and protein levels of CB2R in the dorsal root ganglia and paw as well as of NOS1 in the dorsal root ganglia of WT mice, with and without peripheral inflammation, were also assessed. Finally, to evaluate if the cGMP-PKG-KATP peripheral pathway activation could modulate the local effects produced by JWH-015, the antiallodynic and antihyperalgesic effects produced by this agonist co-administered with different doses of a selective soluble guanylate cyclase (1H-[1], [2], [4]oxadiazolo[4,3-a]quinoxalin-1-one; ODQ) or a PKG ((Rp)-8-(para-chlorophenylthio)guanosine-3′,5′-cyclic monophosphorothioate; Rp-8-pCPT-cGMPs) inhibitor as well as by a selective KATP channel blocker (glibenclamide), were also determined.

## Results

### Effects of the subplantar administration of JWH-015 in the mechanical allodynia and thermal hyperalgesia induced by CFA

**Figure 1:**
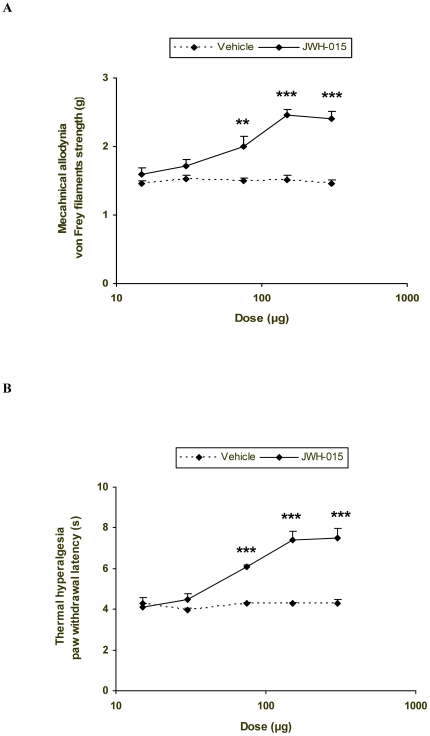
The antiallodynic and antihyperalgesic effects of JWH-015. Effects of the subplantar administration of different doses (logarithmic axis) of JWH-015 or vehicle on the mechanical allodynia (A) and thermal hyperalgesia (B) induced by CFA, in the ipsilateral paw of WT mice at 10 days after their injection. JWH-015 was administered 20 min before starting behavioral testing. Data are expressed as mean values of the von Frey filaments strength (g) for mechanical allodynia and as the paw withdrawal latency (s) for thermal hyperalgesia ± SEM (5–6 animals per dose). In both tests, for each dose, ** p<0.01 and *** p<0.001 denotes significant differences between JWH-015 and vehicle treated animals (Student's t test).

In a mouse model of CFA-induced inflammatory pain [19], our results show that the subplantar administration of JWH-015 into the ipsilateral paw dose-dependently inhibited the mechanical allodynia (Fig. 1A) and thermal hyperalgesia (Fig. 1B) induced by the inflammatory agent. Thus, the mechanical antiallodynic and thermal antihyperalgesic effects produced by different doses of JWH-015 (15-300 µg) in the ipsilateral paws of CFA-injected WT mice were significantly higher than those obtained in their corresponding vehicle treated groups (p<0.01; Student's t test). The subplantar administration of JWH-015 or vehicle did not produce any significant effect on the contralateral paw of these animals (data not shown).

### Reversion of the antinociceptive effects of JWH-015 by AM630, NX-ME or AM251 after chronic inflammatory pain

**Figure 2:**
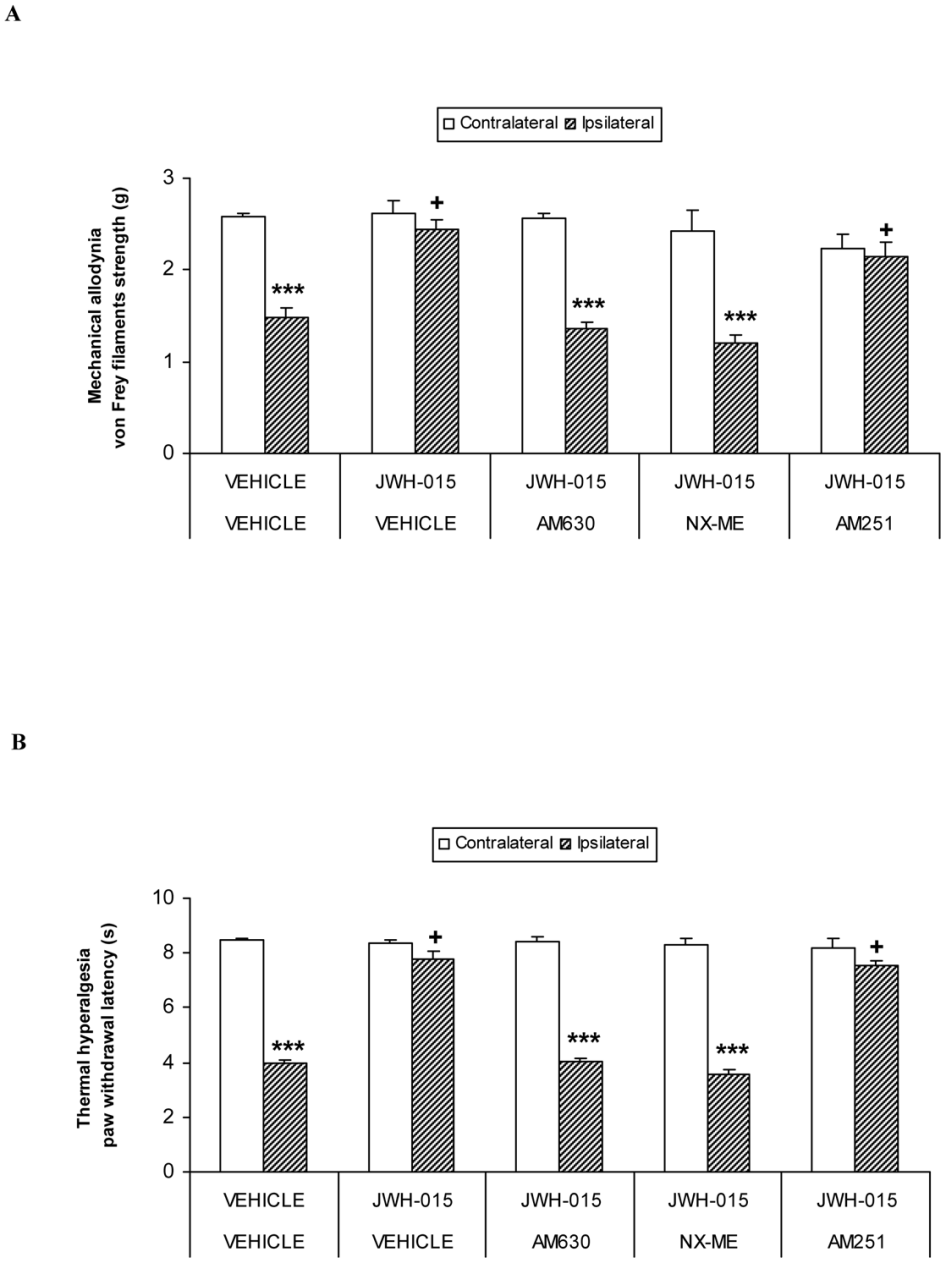
Reversion of the antinociceptive effects of JWH-015. Effects of the subplantar administration of vehicle, AM630 (60 µg), NX-ME (150 µg) or AM251 (150 µg) on the inhibition of the mechanical allodynia (A) and thermal hyperalgesia (B) induced by the subplantar administration of JWH-015 (150 µg) in CFA-injected WT mice. Data are expressed as mean values of the von Frey filaments strength (g) for mechanical allodynia and as the paw withdrawal latency (s) for thermal hyperalgesia ± SEM (5-6 animals per group). For each test and drug, *** indicates significant differences when compared to their corresponding contralateral paw (p<0.001, paired Student's t test) and + indicates significant differences as compared with the ipsilateral paw of the vehicle treated group (p<0.05, one way ANOVA followed by the Student-Newman-Keuls test).

The administration of CFA induced a significant mechanical allodynia (Fig. 2A) and thermal hyperalgesia (Fig. 2B) in the ipsilateral paw as compared to their corresponding contralateral paw (p<0.001; paired Student's t test). The antiallodynic (Fig. 2A) and antihyperalgesic (Fig. 2B) effects produced by a high dose of JWH015 in the ipsilateral paw of CFA-injected WT mice were completely reversed by their subplantar co-administration with a selective CB2R (AM630) or a peripheral opioid receptor (NX-ME) antagonist (p<0.001; paired Student's t test compared to their corresponding contralateral paw). The subplantar administration of AM251 (a selective CB1R antagonist) was unable to revert the local antiallodynic and antihyperalgesic effects produced by JWH-015 (p<0.05; one way ANOVA, followed by Student Newman Keuls, compared with the ipsilateral paw of the vehicle treated group). The subplantar administration of the agonist alone or combined with the different tested antagonists did not produce any significant effect in the contralateral paw as compared to vehicle. In addition, the subplantar administration of AM630, NX-ME, AM251 or vehicle alone in CFA-injected mice did not show any significant effect on the two different nociceptive responses evaluated in this study (data not shown).

### The mRNA and protein levels of CB2R in the dorsal root ganglia and paw of WT mice with and without chronic inflammatory pain

**Figure 3:**
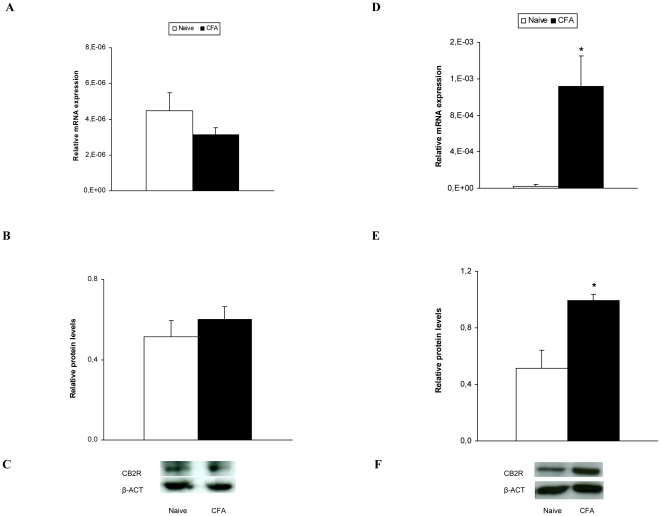
Dorsal root ganglia and paw expression of CB2R. The relative mRNA (A and D) and protein (B and E) expression of CB2R in the ipsilateral side of the dorsal root ganglia (left) and paw (right) from naive and CFA injected WT mice were shown. A representative example of Western blots for CB2R protein (37 kDa) in which β-actin (43 kDa) was used as a loading control is shown in C and F. Data are expressed as mean values ± SEM; n = 4–5 samples per group. * indicates significant differences when compared CFA vs. naïve mice (p<0.03, Student's t test).

The mRNA and protein levels of CB2R in the ipsilateral side of the dorsal root ganglia (A and B) and paw (D and E) from WT mice, with (CFA) and without (naive) inflammatory pain, are shown in Figure 3. While peripheral inflammation did not alter the dorsal root ganglia mRNA and protein expression of CB2R, it significantly increased their expression in the paw (p<0.030 Student's t test as compared to naive animals).

### The mRNA levels and protein levels of NOS1 in the dorsal root ganglia of WT mice with and without chronic inflammatory pain

**Figure 4:**
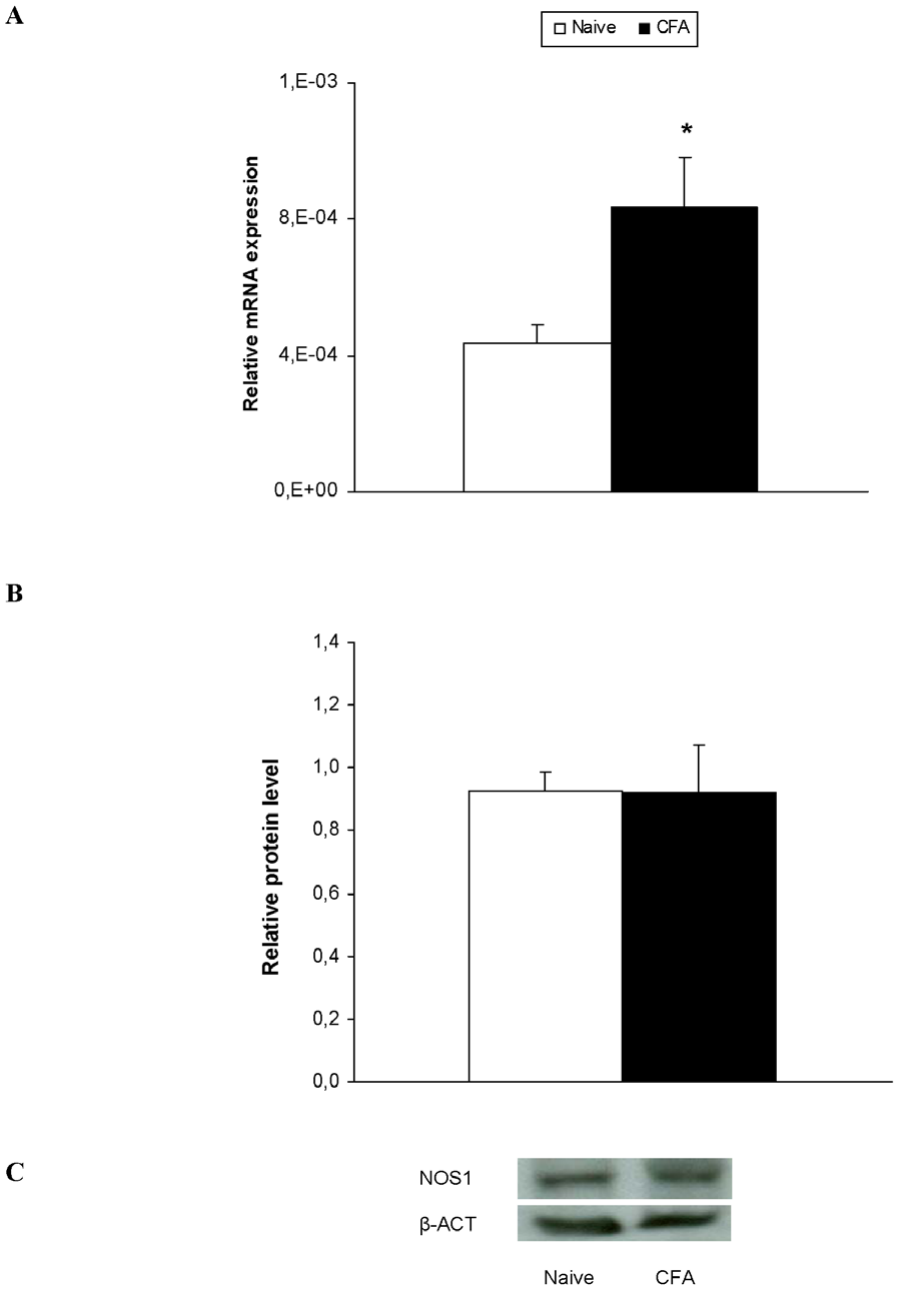
Dorsal root ganglia expression of NOS1. The relative mRNA (A) and protein (B) expression of NOS1 in the ipsilateral side of the dorsal root ganglia from naive and CFA injected WT mice are represented. A representative example of Western blot for NOS1 protein (155 kDa) in which β-actin (43 kDa) was used as a loading control is shown in C. Data are expressed as the mean values ± SEM of 5–6 samples per group. * indicates significant differences when compared CFA vs. naïve mice (p<0.05, Student's t test).

The mRNA and protein levels of NOS1 in the ipsilateral side of the dorsal root ganglia from WT mice with (CFA) and without (naive) inflammatory pain are shown in Figure 4A and 4B, respectively. Our results showed that inflammatory pain significantly enhanced the mRNA expression of NOS1, but not their protein levels, in the ipsilateral side of CFA injected mice as compared to naive (p<0.032, Student's t test).

### The role of nitric oxide synthesized by NOS1 in the local antinociceptive effects produced by JWH-015 during chronic inflammatory pain

**Figure 5:**
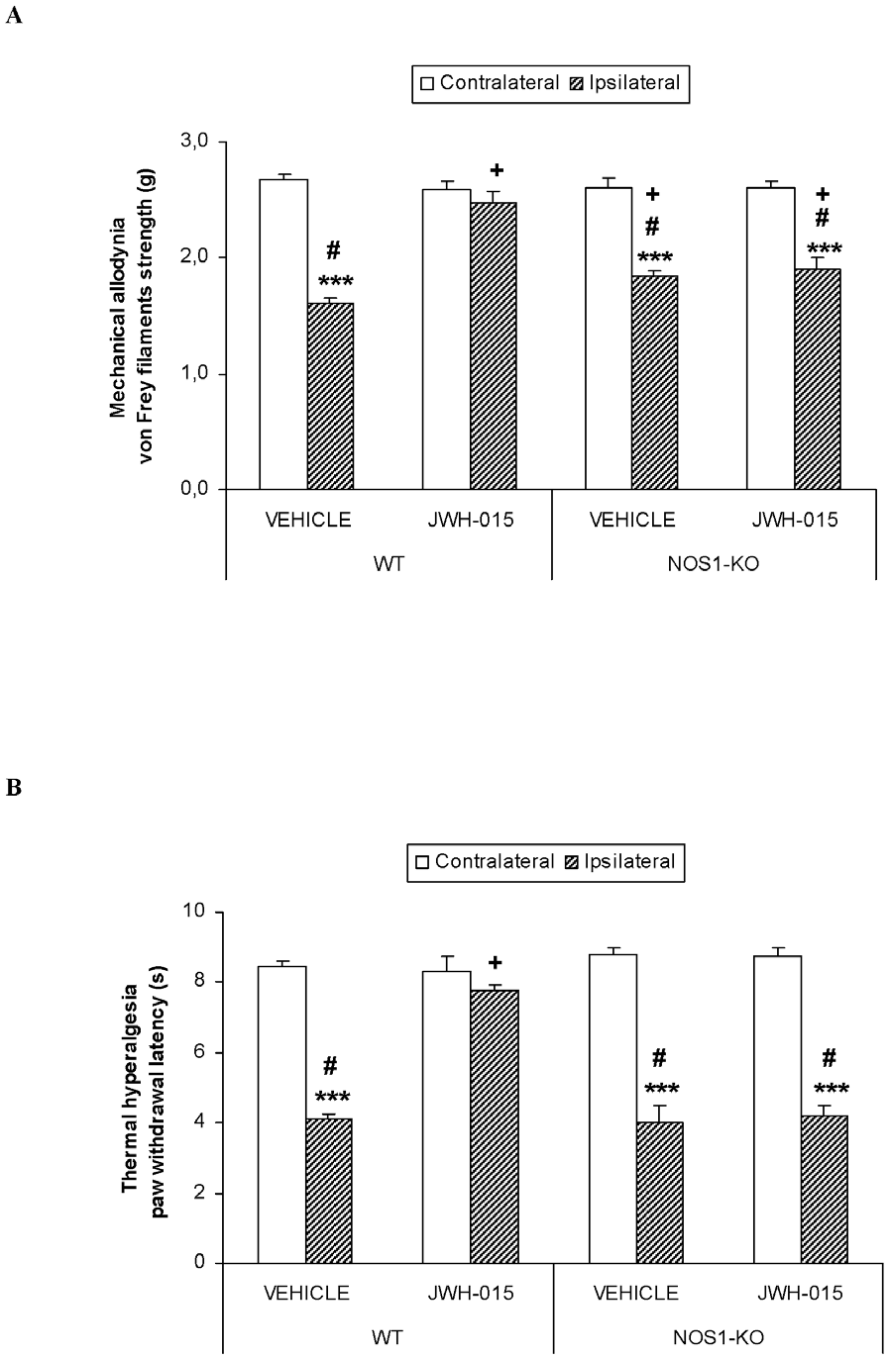
The antiallodynic and antihyperalgesic effects of JWH-015 in WT and NOS1-KO mice. Effects of the subplantar administration of 150 µg of JWH-015 or vehicle in the contralateral and ipsilateral paw withdrawal latencies to a mechanical (A) or thermal (B) stimulus in WT and NOS1-KO mice, at 10 days after CFA injection. Data are expressed as mean values of the von Frey filaments strength (g) for mechanical allodynia and as the paw withdrawal latency (s) for thermal hyperalgesia ± SEM (5–6 animals per group). For each test, *** p<0.001, indicates significant differences vs. their contralateral paw (paired Student's t test), + indicates significant differences as compared with the ipsilateral paw of vehicle treated group (p<0.05, one way ANOVA followed by the Student-Newman-Keuls test) and # indicates significant differences as compared with the ipsilateral paw of WT group treated with JWH-015 (p<0.05, one way ANOVA followed by the Student-Newman-Keuls test).

The role played by nitric oxide synthesized by NOS1 in the local antinociceptive effects produced by JWH-015 during peripheral inflammation was evaluated by comparing the antiallodynic (Fig. 5A) and the antihyperalgesic (Fig. 5B) effects produced by a high dose of this agonist (150 µg) in WT and NOS1-KO mice at 10 days after CFA injection. Our results show that the subplantar injection of CFA induces a reduced mechanical allodynia (p<0.05; one way ANOVA, followed by Student Newman Keuls test) and a similar thermal hyperalgesia in the ipsilateral paw of NOS1-KO mice as compared to WT. The subplantar administration of JWH-015 only reversed these effects in WT mice, but not in NOS1-KO (p<0.001, paired Student's t test, comparing ipsilateral vs. contralateral paw). Moreover, the effects produced by JWH-015 in the ipsilateral paw of NOS1-KO mice are significantly lower to that those produced by this drug in the ipsilateral paw of WT mice (p<0.05; one way ANOVA, followed by Student Newman Keuls test). In both genotypes the subplantar administration of JWH-015 or vehicle did not have any significant effect on the contralateral paw of these animals.

### Involvement of the peripheral nitric oxide–cGMP–PKG-KATP signaling pathway in the local antiallodynic and antihyperalgesic effects produced by JWH-015 during chronic inflammatory pain

The role of the peripheral nitric oxide-cGMP-PKG-KATP signaling pathway in the local mechanical antiallodynic and thermal antihyperalgesic effects produced by JWH-015 in CFA-injected WT mice was assessed by evaluating the effects produced by 150 µg of this agonist co-administered with different dose of ODQ, Rp-8-pCPT-cGMPs or glibenclamide.

**Figure 6:**
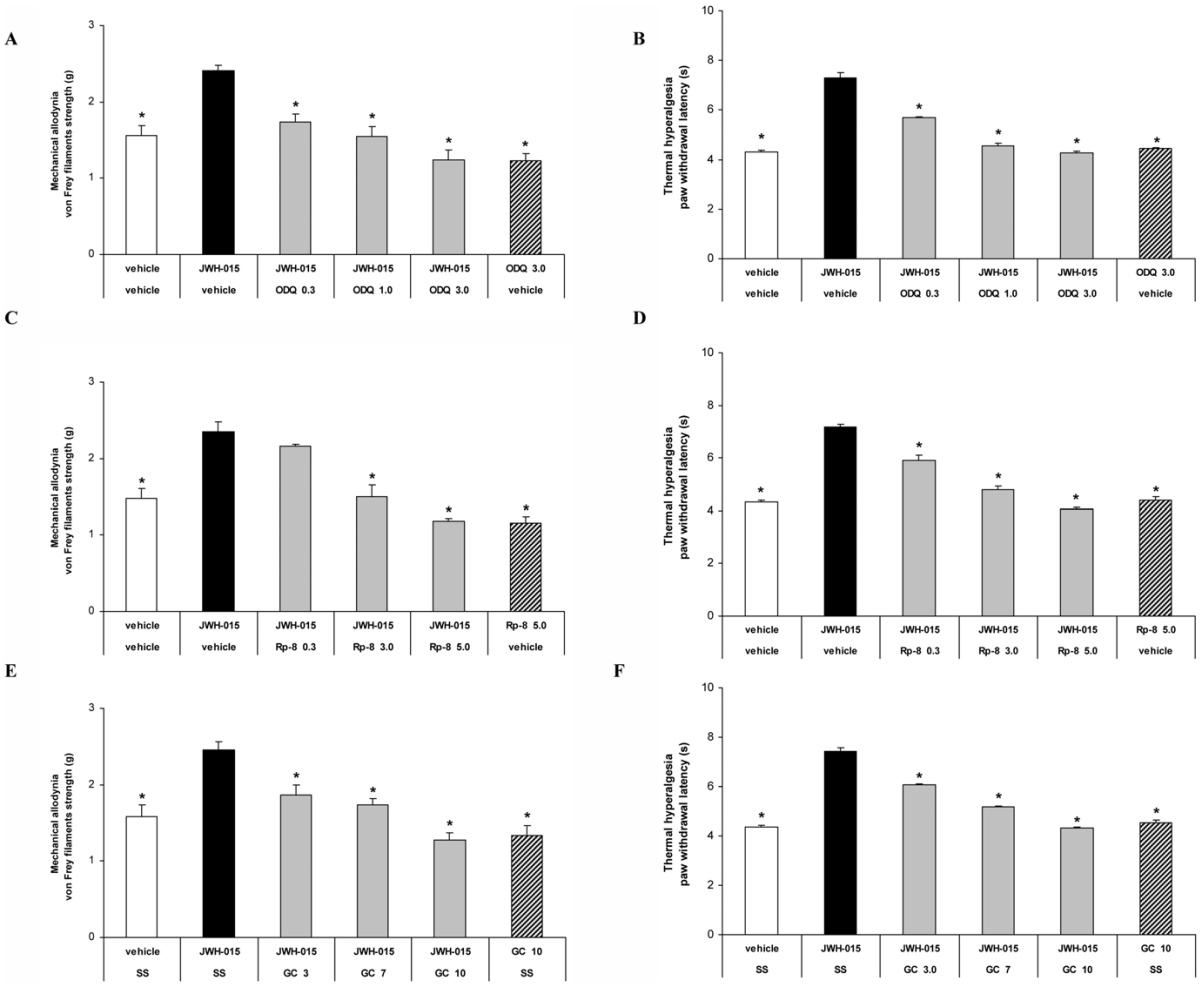
The role of the peripheral nitric oxide–cGMP–PKG-KATP signaling pathway in the antinociceptive effects of JWH-015. Mechanical antiallodynic (A, C, E) and thermal antihyperalgesic (B, D, F) effects of the subplantar co-administration of JWH-015 (150 µg) plus vehicle or different doses of ODQ (0.3 – 3.0 µg; A, B), Rp-8-pCPT-cGMPs (Rp-8; 0.3-5.0 µg; C, D) or glibenclamide (GC; 3.0–10 µg; E,F) in the ipsilateral paw of WT mice at 10 days after CFA injection. The effects of the subplantar administration of vehicle and the maximal doses of ODQ (3.0 µg), Rp-8 (5.0 µg) or glibenclamide (10.0 µg) administered alone are also shown. All drugs were administered 20 min before starting behavioral testing. Data are expressed as mean values of the von Frey filaments strength (g) for mechanical allodynia and as the paw withdrawal latency (s) for thermal hyperalgesia ± SEM (5–6 animals per group). For each behavioral test and selective inhibitor assayed, * p<0.05 denotes significant differences vs. group treated with JWH-015 + vehicle (one way ANOVA followed by Student Newman Keuls test).

Our results showed that the local antiallodynic and antihyperalgesic effects produced by JWH-015 in the ipsilateral paw of CFA-injected WT mice were significantly inhibited by their peripheral co-administration with different doses of ODQ (Fig. 6, A-B), Rp-8-pCPT-cGMPs (Fig. 6, C-D) or glibenclamide (Fig. 6, E-F) in a dose-dependent manner (p<0.001, one way ANOVA followed by Student Newman Keuls test). While the local co-administration of JWH-015 plus ODQ, Rp-8-pCPT-cGMPs or glibenclamide did not have any significant effect on the contralateral paw of CFA-injected mice (data not shown). Our results also indicated that the subplantar administration of the highest tested doses of ODQ (3 µg), Rp-8-pCPT-cGMP (5 µg), glibenclamide (10 µg) did not produce any significant antiallodynic or antihyperalgesic effect in the ipsilateral or contralateral paw of CFA-injected WT mice.

## Discussion

In this study, we showed for first time that the local administration of JWH-015 dose-dependently inhibited the mechanical allodynia and thermal hyperalgesia induced by CFA through the activation of the peripheral nitric oxide-cGMP-PKG-KATP channel signaling pathway, triggered by NOS1 and mediated by opioids. Indeed, the local mechanical antiallodynic and thermal antihyperalgesic effects produced by a high dose of JWH-015 were blocked by NX-ME, annulled in NOS1-KO mice and dose-dependently diminished by their co-administration with different doses of ODQ, Rp-8-pCPT-cGMPs and glibenclamide. Our results also show that chronic inflammatory pain increases the paw expression of CB2R as well as to the dorsal root ganglia transcription of NOS1.

Several works demonstrated that the local administration of CB2R selective agonists attenuates the thermal and mechanical hypersensitivity induced by carrageenan or CFA in different models of acute *(hours to two days)* inflammatory pain [2], [8], [20]. Our results support and expand this hypothesis in a chronic model of inflammatory pain at 10 days after CFA injection. The CB2R specificity of the inhibitory effects induced by JWH-015 was demonstrated by the complete reversion of their effects with the local co-administration with a selective CB2R, but not a CB1R, antagonist. In addition, the fact that the highest dose of JWH-015 did not produce any significant effect in the contralateral paw of CFA-injected mice denotes the peripheral site of action of this drug.

Our data also show that although chronic inflammatory pain did not alter the peripheral mRNA or protein levels of CB2R in the dorsal root ganglia, it increases their expression in the paw. This is in accordance with the unchanged expression of these receptors observed in the dorsal root ganglia of animals with bone-cancer induced chronic pain [3] as well as to the increased expression of those observed in the paw of animals with acute inflammatory pain [8]. Thus, our results support these data and expand theme to chronic inflammatory pain conditions.

It is known that the antinociceptive effects produced by a specific CB2R agonist (AM1241) are mediated through the release of β-endorphins which appear to act at µ-opioid receptors located on the terminals of primary afferent neurons to produce peripheral antinociception during acute inflammation and bone cancer pain [3], [17]–[18]. Our results demonstrated that the antiallodynic and antihyperalgesic effects produced by JWH-015 were completely reversed by their local co-administration with a peripherally acting opioid receptor antagonist. These findings revealed that during chronic inflammatory pain the opioid-mediated antinociception derived from the activation of peripheral CB2R by JWH-015 is also functional.

In accordance with the literature [21], our results also demonstrated that chronic inflammatory pain induced a modest increase in the dorsal root ganglia transcription of NOS1, which did not correlate with an increased protein expression probably related to the much higher sensitivity of the real-time PCR assay compared to the western blot. Several works have been demonstrated that the local antinociceptive effects produced by µ-opioid receptor agonists during inflammation are mainly mediated by the release of nitric oxide synthesized by NOS1 [19], [22]. Thus, and taking account that JWH-015 produces their antinociceptive effects by the activation of peripheral opioid receptors, we have evaluated if this opioid-mediated antinociception induced by CB2R activation is also produced via NOS1 by using knockout mice. The fact that the local administration of JWH-015 did not block the mechanical and thermal hypersensitivity induced by CFA in NOS1-KO animals suggests that nitric oxide synthesized by NOS1 also participates in the local antinociceptive effects produced by this agonist during chronic inflammatory pain.

The possible involvement of the peripheral cGMP-PKG-KATP channel signaling pathway in the local effects of a CB2R agonist after chronic inflammatory pain was also evaluated. Interestingly, and in contrast to neuropathic pain [16], the local pharmacological blockage of the nitric oxide-cGMP-PKG-ATP signaling pathway diminished the peripheral antiallodynic and antihyperalgesic effects of a CB2R agonist after CFA injection. That is, the inhibitory effects induced by JWH-015 were dose-dependently diminished by their peripheral co-administration with ODQ, Rp-8-pCPT-cGMPs or glibenclamide. Therefore, and similarly to what occurs with a CB1 endocannabinoid [15], the peripheral analgesia induced by JWH-015 under chronic inflammatory conditions depends on the activation of the local nitric oxide-cGMP-PKG-KATP channel signaling pathway.

In summary, our data demonstrate that the peripheral nitric oxide-cGMP-PKG-KATP signaling pathway, triggered by NOS1 and mediated by local endogenous opioids, participates in the antinociceptive effects produced by JWH-015 and suggest that the activation of this pathway might be an interesting therapeutic target for the treatment of chronic inflammatory pain with cannabinoids.

## Materials and Methods

### Ethics statement

Animal procedures were conducted in accordance with the guidelines of the European Communities, Directive 86/609/EEC regulating animal research and approved by the local ethical committee of our Institution (Comissió d'Etica en l'Experimentació Animal i Humana de la Universitat Autònoma de Barcelona, #00801).

### Animals

Male NOS1-KO mice (C57BL/6J background) were purchased from the Jackson Laboratory (Bar Harbor, ME, USA), while WT mice with the same genetic background (C57BL/6J) were acquired from Harlan Laboratories (Barcelona, Spain). All mice weighing 21 to 25 g were housed under 12-h/12-h light/ dark conditions in a room with controlled temperature (22°C) and humidity (66 %). Animals had free access to food and water and were used after a minimum of 6 days acclimatization to the housing conditions. All experiments were conducted between 9:00 AM and 5:00 PM.

### Induction of chronic inflammation

Chronic inflammatory pain was induced in WT and NOS1-KO mice by the subplantar injection of 30 µl of complete Freund's adjuvant (CFA; Sigma) into the right hind paw under brief anesthetic conditions with isoflurane according to the method described by Larson et al. [23]. All experiments were performed at 10 days after CFA injection. At this time point, all of these animals developed a local inflammatory reaction, allodynia to mechanical stimuli and hyperalgesia to noxious thermal stimuli as previously reported by our group [19].

### Nociceptive behavioral tests

#### Mechanical allodynia

The mechanical allodynia was quantified by measuring the hind paw withdrawal response to von Frey filament stimulation. In brief, animals were placed in a Plexiglas® box (20 cm high, 9 cm diameter) with a wire grid bottom through which the von Frey filaments (North Coast Medical, Inc., San Jose, CA, USA) bending force range from 0.008 to 3.5 g, were applied by using a modified version of the up–down paradigm, as previously reported by Chaplan et al. [24]. The filament of 0.4 g was used first and the 3.5 g filament was used as a cut-off. Then, the strength of the next filament was decreased or increased according to the response. The threshold of response was calculated from the sequence of filament strength used during the up–down procedure by using an Excel program (Microsoft Iberia SRL, Barcelona, Spain) that includes curve fitting of the data. Clear paw withdrawal, shaking or licking of the paw were considered nociceptive-like responses. Both ipsilateral and contralateral hind paws were tested. Animals were allowed to habituate for 1 h before testing in order to allow an appropriate behavioral immobility.

#### Thermal hyperalgesia

The thermal hyperalgesia was assessed as previously reported by Hargreaves et al. [25]. Paw withdrawal latency in response to radiant heat was measured using the plantar test apparatus (Ugo Basile, Italy). Briefly, mice were placed in Plexiglas boxes (20 cm high × 9 cm diameter) positioned on a glass surface. The heat source was positioned under the plantar surface of the hind paw and activated with a light beam intensity, chosen in preliminary studies to give baseline latencies from 8 to 10 s in control mice. A cut-off time of 12 s was used to prevent tissue damage in absence of response. The mean paw withdrawal latencies from the ipsilateral and contralateral hind paws were determined from the average of 3 separate trials, taken at 5 min intervals to prevent thermal sensitization and behavioral disturbances. Animals were habituated to the environment for 1 h before the experiment to become quiet and to allow testing.

### Molecular experiments

#### Tissue isolation

Animals were sacrificed at 0 (naïve) and 10 days after CFA-injection by cervical dislocation. Three ganglia from the lumbar section (L3 to L5) of the ipsilateral site from WT and NOS1-KO mice were removed immediately after sacrifice, frozen in liquid nitrogen and stored at −80°C until assay. Samples from four to five animals were pooled together to obtain enough RNA or protein levels for performing the real time-PCR or Western blot analysis, respectively. In these experiments naïve mice that did not receive any injection were used as controls.

#### Total RNA extraction and reverse transcription

Tissues were homogenized in ice-cold with a homogenizer (Ultra-Turf, T8; Ika Werke, Staufen, Germany) and the total RNA was extracted with TRIzol reagent (Invitrogen, Renfrewshire, England). The amount of the purified RNA (A_260_/A_280_ ratio was ≥1.9) was determined by spectrophotometry. In all experiments, 1 µg of total RNA was reverse transcribed into cDNA using SuperScript II RNAse H^-^ reverse transcriptase (Invitrogen, Renfrewshire, UK) in a final volume of 10 µl. Negative controls were performed in which all of the components were included except reverse transcriptase.

#### TaqMan probe real-time polymerase chain reaction (PCR)

The expression of NOS1 and CB2R was determined by real-time PCR using the pre-developed mice TaqMan® gene expression assay: Mm0435189_m1 for NOS1 and Mm00438286_m1 for CB2R (Applied Biosystems, CA, USA). A probe against GAPDH (Mm 99999915_g1) was used as endogenous control and reactions without RNA were included as negative controls to ensure the specificity. PCR reactions were set up in 96-well plates containing the corresponding cDNA, 0.9 µmol/L of each forward and reverse primers, 0.25 µmol/L of TaqMan® MGB probe and a final concentration of 1x universal master mix (Applied Biosystems, CA, USA), which provides the PCR buffer, MgCl_2_, dNTPs, and the thermal stable AmpliTaq Gold DNA polymerase. The assay was conducted using the Applied Biosystems ABI PRISM 7000 Sequence Detection System. All samples were assayed in duplicate. Relative expression of the target gene was calculated by means of the comparative threshold cycle method [26].

#### Western blot analysis

The CB2R protein levels in the dorsal root ganglia and paw tissue and the NOS1 protein levels in the dorsal root ganglia were analyzed by Western blot. Tissues were homogenized in buffer (50 mM Tris-Base, 150 nM NaCl, 1% NP-40, 2 mM EDTA, 1 mM phenylmethylsulfonyl fluoride, 0.5 Triton X-100, 0.1% SDS, 1 mM Na_3_VO_4_, 25 mM NaF, 0.5 % protease inhibitor cocktail, 1% phosphatise inhibitor cocktail). All reactive were purchased at Sigma (St. Louis, MO, USA) with the exception of NP-40 from Calbiochem (Biosciences, La Jolla, CA, USA). The crude homogenate was solubilized 1 hour at 4°C, sonicated for 10 seconds and centrifugated at 4°C for 15 min at 700 × g. For the CB2R, 50 µg of total protein, were mixed with 4 × laemmli loading buffer and then loaded onto 4% stacking/10% separating SDS-polyacrylamide gels. The proteins were electrophoretically transferred onto PVDF membrane during 2 hours, blocked with PBS +10% BSA, and subsequently incubated overnight at 4°C with a polyclonal rabbit anti-CB2R antibody (1∶500, Abcam, Cambridge, UK). For the NOS1, 100 µg of total protein, were mixed with 4 × laemmli loading buffer and then loaded onto 4% stacking/5% separating SDS-polyacrylamide gels. The proteins were electrophoretically transferred onto PVDF membrane overnight, blocked with PBS +10% BSA, and subsequently incubated overnight at 4°C with a polyclonal rabbit anti-NOS1 antibody (1∶100, BD Transduction, BD Transduction Laboratories, San Diego, CA, USA).

The proteins were detected by an horseradish peroxidase-conjugated anti-rabbit secondary antibody (GE Healthcare, Little Chalfont, Buckinghamshire, UK) and visualized by chemiluminescence reagents provided with the ECL kit (Amersham Pharmacia Biotech, Piscataway, NJ, USA) and exposure onto hyperfilm (GE, Healthcare). Membranes were stripped and reproved with a monoclonal rabbit anti-β-actin antibody (1∶10.000, Sigma, St. Louis, MO, USA). β-actin was used as a loading control. The intensity of blots was quantified by densitometry.

### Experimental protocol

In a model of CFA-induced inflammatory pain in mice [19], we investigated the mechanical antiallodynic and thermal antihyperalgesic effects of the subplantar administration of various doses of a selective CB2R agonist, JWH-015 (15–300 µg) or vehicle in the ipsilateral and contralateral paw of WT mice at 10 days after CFA injection. All animals were tested in each paradigm at pre and post drug administration. The specificity and the possible participation of the endogenous opioids in the local antinociception produced by a high dose (150 µg) of JWH-015 was assessed by evaluating the reversibility of their effects with the peripheral co-administration of 60 µg of AM630 (a selective CB2R antagonist), 150 µg of AM251 (a selective CB1R antagonist) or 150 µg of NX-ME (a peripheral opioid receptor antagonist). This dose of JWH-015 was selected based upon their high efficacy in inhibiting the mechanical allodynia and thermal hyperalgesia induced by peripheral inflammation and the doses of the antagonists according to previous studies in the literature [2]–[3], [27]. The mRNA and protein levels of CB2R in the dorsal root ganglia and paw as well as of NOS1 in the dorsal root ganglia from the ipsilateral site of naïve and CFA-injected WT mice by using real time PCR and Western blot analysis, were also assessed. In another set of experiments, the involvement of nitric oxide synthesized by NOS1 in the local antinociceptive effects of JWH-015 during peripheral inflammatory pain was investigated by using knockout mice. Thus, the effects of the subplantar administration of 150 µg of JWH-015 or vehicle on the mechanical allodynia (von Frey filaments) and thermal hyperalgesia (plantar test) induced by peripheral inflammation in NOS1-KO mice, were also evaluated. Finally, the possible involvement of the peripheral nitric oxide-cGMP-PKG-KATP signaling pathway in the local mechanical and thermal antinociceptive effects of JWH-015 was also evaluated. For this purpose in WT mice at 10 days after CFA injection, the local antinociceptive effects produced by a high dose of JWH-015 (150 µg) combined with different doses of ODQ (0.3–3.0 µg) a selective soluble guanylate cyclase inhibitor [28], Rp-8-pCPT-cGMPs (0.3–5.0 µg) a PKG inhibitor [29] or glibenclamide (3.0–10.0 µg) a KATP channel blocker [30], selected according to previous studies in the literature [31], were also determined.

### Drugs

JWH-015, AM630 and AM251 were obtained from Tocris (Ellisville, MI). ODQ, Rp-8-pCPT-cGMPs, glibenclamide and NX-ME were purchase from Sigma-Aldrich (St. Louis, MO). JWH-015 and ODQ were dissolved in dimethyl sulfoxide (DMSO) at 10% solution in saline while AM630, AM251 and glibenclamide were dissolved in DMSO at 50% solution in saline. NX-ME and Rp-8-pCPT-cGMPs was dissolved in saline solution (0.9% NaCl). All drug combinations were diluted in the highest required concentration of DMSO. All drugs alone or combined were injected in a final volume of 30 µl. In all experiments, drugs were administered into the plantar side of the right paw, 20 min before behavioral testing. For each group treated with a drug the respective control group received the same volume of vehicle.

### Statistical analysis

Data are expressed as mean ± standard error of the mean (SEM). For each test, the comparison of the effects produced by different doses of JWH-015 in the contralateral and ipsilateral paws was compared to the effects produced by vehicle in the same paw by using a Student's t test. For each test, the reversion of the antinociceptive effects produced by JWH-015 in the ipsilateral and contralateral paws of WT mice was analyzed by comparing the effects produced by the subplantar administration of vehicle, JWH-015 alone or co-administered with AM630, NX-ME or AM251 by using a one-way ANOVA followed by the Student-Newman-Keuls test. The comparison of the effects produced by the different treatments in the contralateral and ipsilateral paws of CFA-injected mice was evaluated by using a paired Student's t test.

Changes in the expression of NOS1 and CB2R in the ipsilateral sites of the dorsal root ganglia and/or paw from naïve and CFA-injected WT mice were analyzed by using a Student's t test.

For each genotype, the analysis of the antinociceptive effects produced by a high dose of JWH-015 or vehicle in the ipsilateral paw as compared to their corresponding contralateral paw was performed by using a paired Student's t test. For each paw, the comparison of the effects produced by JWH-015 or vehicle in WT and NOS1-KO mice was evaluated by using a one way ANOVA followed by Student Newman Keuls test. The comparison of the antiallodynic and antihyperalgesic effects produced by a high dose of JWH-015 subplantarly administered alone or combined with different doses of selective inhibitors (ODQ and Rp-8-pCPT-cGMPs) or a blocker (glibenclamide) in the contralateral and ipsilateral paws of CFA-injected WT mice was performed by using a one way ANOVA followed by the Student Newman Keuls test.

A value of p<0.05 was considered as a significant.
